# Enhancing Healthcare Access–Smartphone Apps in Arrhythmia Screening: Viewpoint

**DOI:** 10.2196/23425

**Published:** 2021-08-27

**Authors:** Marcin Książczyk, Agnieszka Dębska-Kozłowska, Izabela Warchoł, Andrzej Lubiński

**Affiliations:** 1 Department of Interventional Cardiology and Cardiac Arrhythmias Medical University of Lodz Łódź Poland; 2 Department of Noninvasive Cardiology Medical University of Lodz Łódź Poland

**Keywords:** arrhythmia screening, atrial fibrillation, mobile electrocardiography, mobile health, phonocardiography, photoplethysmography, seismocardiography, stroke prevention

## Abstract

Atrial fibrillation is the most commonly reported arrhythmia and, if undiagnosed or untreated, may lead to thromboembolic events. It is therefore desirable to provide screening to patients in order to detect atrial arrhythmias. Specific mobile apps and accessory devices, such as smartphones and smartwatches, may play a significant role in monitoring heart rhythm in populations at high risk of arrhythmia. These apps are becoming increasingly common among patients and professionals as a part of mobile health. The rapid development of mobile health solutions may revolutionize approaches to arrhythmia screening. In this viewpoint paper, we assess the availability of smartphone and smartwatch apps and evaluate their efficacy for monitoring heart rhythm and arrhythmia detection. The findings obtained so far suggest they are on the right track to improving the efficacy of early detection of atrial fibrillation, thus lowering the risk of stroke and reducing the economic burden placed on public health.

## Introduction

The most commonly reported arrhythmia is atrial fibrillation (AF) [1]. Its prevalence is still underestimated [2], particularly the asymptomatic form: silent AF. Even so, the prevalence of symptomatic AF is estimated to be 0.12%-0.16% in patients aged <49 years, 3.7%-4.2% in patients aged 60-70 years, and almost 10%-17% in those aged ≥80 years [3]. The most common undiagnosed and untreated AF complications are thromboembolic events, such as stroke, which occur up to 5.6 times more frequently in AF patients [4]. It is therefore desirable to provide screening to patients in order to detect atrial arrhythmias. Additionally, the European Society of Cardiology (ESC) 2020 ESC Guidelines for the diagnosis and management of AF recommends opportunistic screening for AF by pulse taking or electrocardiogram (ECG) rhythm strip in patients above 65 years of age, and systematic ECG screening in patients above 75 years of age or those at high risk of stroke [5]. A problem arises when occasionally performed ECG does not record any arrhythmia, and the patient demonstrates palpitations or even worse symptoms, such as a thromboembolic event. As the prevalence of silent AF is estimated to be 10%-25% in the general population [6] and 30%-44% in older adults [7], it is reasonable to promote active screening for AF in patients at risk of the disease. 

Specific mobile apps and accessory devices, such as smartphones and smartwatches, may play a significant role in monitoring heart rhythm in populations who are at high risk of arrhythmia: almost 2.71 billion smartphones are currently in use [8], and almost 150 million smartwatches are predicted to be in use in 2021 [9,10]. In general, the algorithms used by the apps correctly detect AF; however, if an automatic algorithm improperly classifies a trace as AF, it can then be verified and reclassified by a clinician.

In this viewpoint paper, we assess the availability of smartphone and smartwatch apps and evaluate their efficacy for monitoring heart rhythm and arrhythmia detection. These apps are becoming increasingly common among patients and professionals as a part of mobile health (mHealth) [11].

## Methods of Screening for Arrhythmias and Heart Rhythm Monitoring

**Figure 1:**
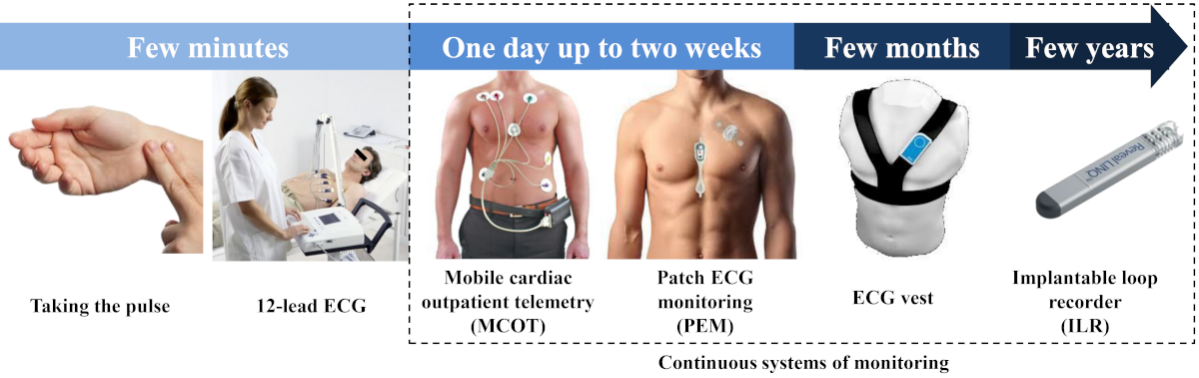
Standard systems of heart rhythm monitoring [12-17]. ECG: electrocardiogram; ILR: implantable loop recorder; MCOT: mobile cardiac outpatient telemetry; PEM: patch electrocardiogram monitoring.

Practically, heart rhythm is typically monitored using continuous and intermittent systems. Continuous systems record the heart rhythm continuously from 24 hours up to 3 years; these show ECG varying in duration or with different numbers of presented leads (Figure 1). 

In contrast, intermittent systems are easily accessible and may play a role when continuous monitoring fails or is unacceptable by the patient. They record the heart rhythm on demand and are typically used upon symptom occurrence or according to a routine schedule (ie, each morning). For this paper, intermittent systems are classified into 5 main groups: standalone devices (ie, MyDiagnostick [18], The Heart Check PEN [19], or Lohman Afib Alert [20]), smartphone/smartwatch apps not dependent on an accessory device, smartphone apps dependent on an accessory device, and smartwatch apps.

The apps created for heart rhythm monitoring record the signal by either photoplethysmography (PPG), electrocardiography (ECG), seismocardiography (SCG), or phonocardiography (PCG). Of these, PPGs and ECGs have achieved commercial success. Some apps have been cleared by the United States Food and Drug Administration (FDA) or certified with the Conformité Européenne (CE) mark. They differ regarding their availability for particular mobile operating systems, duration of sample recording, and their ability to detect irregularity or even differentiate AF from normal sinus rhythm or other arrhythmias.

## Methods for Identifying Available Apps

The European (Poland) App Store and Google Play were searched by 2 independent reviewers (MK and IW) for mobile apps that monitor heart rate. The search was performed between the September 9 and September 16, 2019. The apps offered in the App Store were searched using an iPhone 7 Plus with iOS 12.4.1 (Apple Inc), while those offered in Google Play were searched using a Samsung Galaxy S6 (Samsung Electronics) with Android Oreo 8.1 (Google). The following search string was employed: “heart rate” OR “atrial fibrillation” OR “ECG”. The inclusion criterion comprised the presence of an analogous or automatic algorithm for arrhythmia detection; no exclusion criteria were applied.

## The Overview of Various Technologies and Apps

A total of 7 Android or iOS accessory device–independent smartphone or smartwatch apps, 8 Android or iOS smartphone accessory device–dependent apps, and 4 Android Wear/watchOS smartwatch apps were identified. An accessory device is defined as a tool with at least 2 built-in electrodes which wirelessly connects to a smartphone and is managed from a dedicated app. In addition to “core” apps that were identifiable in the search (Cardiio Pulsometer, Preventicus Heartbeats, and Kardia Mobile), 4 selected “mother-derived” apps were also evaluated: Cardiio Rhythm, Preventicus Nightwatch (both not available commercially), Kardia Band, and Kardia Mobile 6L. Information about these mother-derived apps are available on the developer's website. Due to the prevalence of ECG-based testing among the apps, 2 ECG-based smartphone apps (Kardia and Istel ECG) and 1 smartwatch app (Health) are presented as representative cases. All identified apps and their characteristics are presented in Tables 1 and 2 .

**Table 1:** Characteristics of apps used for heart rhythm monitoring.

App		Mobile operating system	Ratings	Downloads, n^a^	Cost (US $)	Method of recording
**Accessory device–independent apps**						
	Heart_Rhythm	iOS	2.2/5.0	N/A^b^	Free at all	PPG^c^
	Photo Afib Detector	iOS	2.0/5.0	N/A	Free at all	PPG
	Cardiio: Pulsometr^d^	iOS	4.7/5.0	N/A	Free up to 9.99 per month	PPG
	Cardiio Rhythm^e^	iOS	N/A	N/A	N/A	PPG
	Preventicus Heartbeats	Android or iOS	4.1/5.0 4.3/5.0	100,000+	Free up to 43.99 per year	PPG
	FibriCheck	Android or iOS	3.9/5.0 4.6/5.0	100,000+	4.71 up to 12.96 per month	PPG
	Heart Beat	Android	3.5/5.0	10,000+	Free	PCG
	BeatScanner	iOS	2.0/5.0	N/A	Free	SCG^f^
**Accessory device–dependent apps**						
	Kardia Mobile^g^	Android or iOS	3.6/5.0 4.8/5.0	100,000+	Free^h^	ECG^i^
	Kardia Mobile 6L	Android or iOS	3.6/5.0 4.8/5.0	100,000+	Free	ECG
	Kardia Band for Apple Watch Series 1-3	iOS	3.6/5.0 4.8/5.0	100,000+	Free	ECG
	ECG Check	Android or iOS	3.1/5.0 2.6/5.0	10,000+	Free	ECG
	Istel ECG	Android or iOS	4.3/5.0 5.0/5.0	10,000+	Free	ECG
	CardioSecur Pro	Android or iOS	3.4/5.0 4.3/5.0	5000+	Free	ECG
	Sanket Life-ECG, Stress, Fitness	Android or iOS	3.2/5.0 3.3/5.0	1000+	Free	ECG
	GEMS Mobile ECG for HeartCheck CardiBeat	Android or iOS	N/A 5.0/5.0	5000+	Free	ECG
	Coala Heart Monitor	Android or iOS	4.0/5.0 3.9/5.0	1000+	Free	ECG
	i2Dtx for CardioSleeve	iOS	5.0/5.0	N/A	Free	ECG
**Smartwatch apps**						
	Preventicus Nightwatch^e^	Android Wear or watchOS	N/A	N/A	Free	PPG
	FibriCheck	Fitbit OS	N/A	N/A	Free	PPG
	Heart for Apple Watch: All series	watchOS	N/A	N/A	Free	PPG
	ECG app for Apple Watch: Series 4 and subsequent	watchOS	N/A	N/A	Free	ECG
	Huawei Health for Huawei Watch GT	Android Wear	N/A	N/A	Free	PPG
	Heart Health for Garmin Watches	Android Wear	N/A	N/A	Free	PPG

^a^Data available only for Android apps.

^b^N/A: not applicable.

^c^PPG: photoplethysmography.

^d^Formerly known as Cardiio – Heart Rate.

^e^Not available commercially, study version only.

^f^SCG: seismocardiography.

^g^Formerly known as AliveCor.

^h^Device cost not included.

^i^ECG: electrocardiogram.

**Table 2:** Additional characteristics of apps used for heart rhythm monitoring.

App		Automatic irregularity or AF^a^ detection algorithm	FDA^b^ clearance	CE^c^ certificate	Duration of recording	Number of leads if applicable
**Accessory device–independent apps**						
	Heart_Rhythm	No	No	No	10 s	N/A^d^
	Photo Afib Detector	Yes	No	No	30, 60, or 120 s	N/A
	Cardiio: Pulsometr^e^	No	No	No	20 s	N/A
	Cardiio Rhythm^f^	Yes	No	No	20 s	N/A
	Preventicus Heartbeats	Yes	No	Ila	60 or 300 s	N/A
	FibriCheck	Yes	Yes	Ila	60 s	N/A
	Heart Beat	No	No	No	30 s	N/A
	BeatScanner	Yes	No	No	120 s	N/A
**Accessory device–dependent apps**						
	Kardia Mobile^g^	Yes	Yes	Ila	30 s	1
	Kardia Mobile 6L	Yes	Yes	Ila	30 s	6
	Kardia Band for Apple Watch Series 1-3	Yes	Yes	Ila	35 s	1
	ECG Check	Yes	Yes	Ila	45 s	1
	Istel ECG	Yes	No	Ila	30, 60, 120 or 180 s	6
	CardioSecur Pro	Yes	No	Ila	30 s	6-12
	Sanket Life-ECG, Stress, Fitness	No	No	Ila	20 s	1
	GEMS Mobile ECG for HeartCheck CardiBeat	Yes	Yes	Ila	30-300 s	1
	Coala Heart Monitor	Yes	Yes	Ila	60 s	2
	i2Dtx for CardioSleeve	Yes	Yes	Ila	30 s	3
**Smartwatch apps**						
	Preventicus Nightwatch^f^	Yes	No	Ila	Continuous	N/A
	FibriCheck	Yes	Yes	Ila	60 s	N/A
	Heart for Apple Watch: All series	No	No	Ila	Dependent on user activity	N/A
	ECG app for Apple Watch: Series 4 and subsequent	Yes	Yes	Ila	30 s	1
	Huawei Health for Huawei Watch GT	Yes	No	No	Dependent on user activity	N/A
	Heart Health for Garmin Watches	Yes	No	No	Dependent on user activity	N/A

^a^AF: atrial fibrillation.

^b^FDA: Food and Drug Administration.

^c^CE: Conformité Européenne.

^d^N/A: not applicable.

^e^Formerly known as Cardiio – Heart Rate.

^f^Not available commercially, study version only.

^g^Formerly known as AliveCor.

### Apps Using PPG

**Figure 2:**
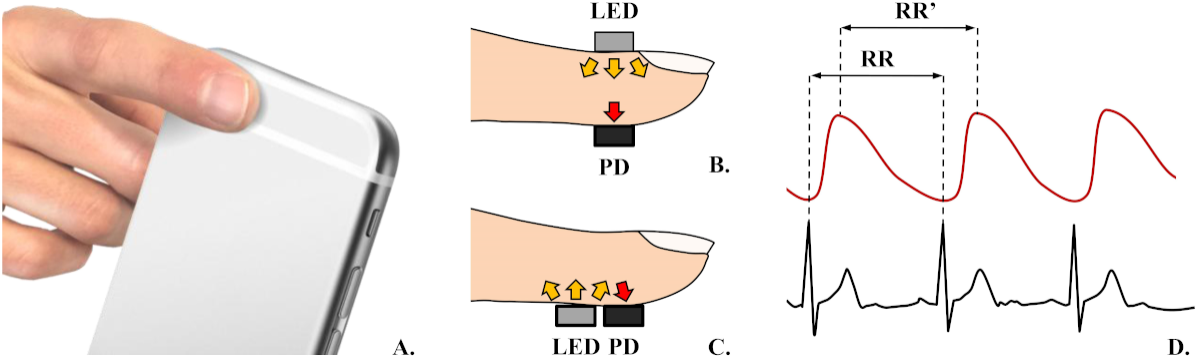
(A) Measuring heart rate with mobile photoplethysmography. The finger is placed over the camera when the flash is on. (B) Transmissive method of measuring heart rate with PPG (used in pulse oximeters). (C) Reflective method of measuring heart rate with photoplethysmography (used in smartphones or smartwatches). (D) Differences in wave shape and RR to RR shift between photoplethysmogram (red curve) and electrocardiogram (black curve) [23-25]. LED: light-emitting diode; PD: photodetector; RR: the interval between 2 Rs in 1 heart cycle.

PPG is a technology in which a light source, such as an light-emitting diode, illuminates a tissue, and a photodetector measures the amount of backscattered light returned [21]. The amount of backscattered light corresponds with the variations of blood volume over the sampling area. As blood volume is synchronous with heartbeat, PPG can accurately show heart rate [22]. Nowadays, it is possible to obtain a photoplethysmogram in a patient suffering from cardiovascular disorders using a smartphone flash acting as a source of light and a camera serving as a photodetector (Figure 2A). Such photoplethysmograms are called “reflective”, as both the light source and photodetector are on the same side of a fingertip. In contrast, systems where the light source and photodetector are located opposite to each other (Figure 2B and C), such as a pulse oximeter, are called “transmissive” [23]. A typical photoplethysmogram wave is shown in red in Figure 2D: its peaks are slightly delayed in relation to the R of the QRS complex in a standard electrocardiogram, representing the time the blood needs to fill up the furthest areas of the body.

#### Heart_Rhythm

Heart_Rhythm is a free app that allows the user to record PPG and then compare the PPG with a model sinus rhythm or atrial fibrillation wave. The efficacy of such subjective self-assessments of rhythm patterns has not been validated in any clinical research [26].

#### Photo AFib Detector

Photo Afib Detector is a free app, which automatically detects an abnormality in the pattern of live-recorded PPG signal by estimating 2 statistical parameters: root mean square of successive difference and Shannon entropy [27]. An algorithm combining root mean square of successive difference and Shannon entropy in an iPhone 4S showed 96.2% sensitivity and 97.5% specificity for beat-to-beat discrimination of AF from sinus rhythm when compared with the 12-lead ECG [28]. However, Photo AFib Detector has not been directly validated in any clinical research.

#### Cardiio: Pulsometer (Former Name: Cardiio–Heart Rate Monitor) and Cardiio Rhythm

Cardiio: Pulsometer is a free app, while Cardiio Rhythm is a beta version currently used only for scientific purposes. 

Cardiio: Pulsometer records high-quality PPG that can be evaluated by an expert and classified as sinus rhythm or rhythm other than sinus; unfortunately, there is no automatic algorithm for arrhythmia detection. Interestingly, the previous version of Cardiio: Pulsometer, called Cardiio – Heart Rate Monitor, was equipped with a face mode that enabled a contactless measurement of the heart rate based on the face of the user. Although Yan et al [29] showed that both finger and face PPGs demonstrate high accuracy in measuring resting heart rates, the app currently only uses the finger mode due to legal reasons [30].

Although Cardiio Rhythm is not currently commercially available, recent clinical findings regarding the app are promising. The sensitivity of the Cardiio Rhythm finger mode (92.9%, 95% CI 77-99) was found to be higher than the internet-enabled mobile ECG distributed by AliveCor (iECG; 71.4, 95% CI 51-87), while Cardiio Rhythm and iECG demonstrated comparable specificity (97.7%, 95% CI 97-99 vs 99.4%, 95% CI 99-100) [31]. Although Cardiio Rhythm demonstrated a lower positive predictive value (PPV) than did iECG (53.1%, 95% CI 38-67 vs 76.9%, 95% CI 56-91), both apps had high negative predictive values (NPV; 99.8%, 95% CI 99-100 vs 99.2, 95% CI 98-100) [31]. Cardiio Rhythm finger mode demonstrated 93.1% sensitivity (95% CI 86.9-97.2) and 90.9% specificity (95% CI 82.9-96.0) compared with superficial ECG, with a 92.2% PPV (95% CI 85.8-95.8) and 92.0% NPV (95% CI 94.8- 95.9) [32]. Finally, Cardiio Rhythm's facial mode effectiveness demonstrated high sensitivity (95%, 95% CI 87-98) and high specificity (96%, 95% CI 91-98) in discriminating AF compared with 12-lead ECG. The PPV and NPV of the facial mode was 92% (95 CI 84-96) and 97% (95% CI 93-99), respectively [33].

#### Preventicus Heartbeats and Preventicus Nightwatch

Preventicus Heartbeats is freely available for smartphones, while Preventicus Nightwatch is available only for smartwatch users. Both apps use PPG in screening for AF, and both have been validated in clinical trials.

The full version of Preventicus Heartbeats allows the user to record PPG and receive a complete report about the rhythm variability. In 2019, the Enhanced Diagnostics for Early Detection of Atrial Fibrillation–Prospective Validation (DETECT AF PRO) trial was performed to compare the efficacy of Preventicus Heartbeats in AF screening with iECG. The sensitivity and specificity of the Preventicus Heartbeats app increased with recording time from 1-3 to 5 minutes: the sensitivity was found to be 89.9% (95% CI 85.5-93.4), 91.3% (95% CI 86.5-94.7), and 91.5% (95% CI 85.9-95.4), respectively, while the specificity was found to be 99.1% (95% CI 97.5-99.8), 98.7% (95% CI 96.7-99.6), and 99.6% (95% CI 97.8-100), respectively [34].

A similar trial, Smartwatches for Detection of Atrial Fibrillation (WATCH AF), was carried out to compare the efficacy of heart rhythm monitoring by the Preventicus Nightwatch smartwatch PPG-based algorithm with that of iECG. One-minute recordings were analyzed by the Preventicus Nightwatch (available for smartwatches only) and compared with the iECG. The algorithm demonstrated 93.7% sensitivity (95% CI 89.8-96.4) and 98.2% specificity (95% CI 95.8- 99.4) in detecting AF [35,36]. Preventicus Nightwatch appear to represent a breakthrough in the monitoring of arrhythmia as it will be able to continuously analyze PPG and document AF events lasting for at least 1 minute. However, it still remains in testing [35].

#### FibriCheck

Fibricheck is the only PPG-based heart rhythm monitoring app cleared both by the FDA and CE. In one study, a comparison of heart rate measurements by FibriCheck and 2 other FDA-cleared devices, Nonin oximeter and AliveCor, found a correlation of 0.834 between FibriCheck and Nonin, 0.88 between FibriCheck and AliveCor, and 0.897 between Nonin and AliveCor (no significant difference; *P*=.61); in addition, an R-R and peak-to-peak interval correlation of 0.993 was found between FibriCheck and wearable ECG (no significant difference; *P*=.92) [37]. 

FibriCheck was also included in the Real Life Digital Population Screening for Atrial Fibrillation Using only a Smartphone (DIGITAL AF II) study, including over 60,000 participants who completed the monitoring period. The study yielded a database of nearly 600,000 pieces of 1-minute PPGs [38]. Of these, 791 participants (1.3%) presented a trace typical for AF. The prevalence of AF in this population was found to be 1.68% in patients aged 40-49 years, 2.16% in those aged 50-59 years, 3.23% in those aged 60-69 years old, 5.97% in those aged 70-79 years, and 12.3% in those aged ≥80 years [38]. Unfortunately, the study has a few limitations: the traces were not compared with any other method, such as iECG or ECG, and only selected data were available. Elsewhere, FibriCheck demonstrated a sensitivity of 96% and a specificity of 91.1% compared with 12-lead ECG [39]. Its cost is not refundable from national health funds [40].

#### Heart for Apple Watch: All Series

The Heart app is an integral part of iOS and watchOS. All Apple Watch series use PPG to record the heart rate, but only series 4 and above are able to record ECG (see section ECG App for Apple Watch: Series 4 or Subsequent). However, Preventicus Nightwatch will be able to use a built-in algorithm to analyze the PPG traces recorded by Apple Watch to detect AF.

### Apps Using Electrocardiography (Dependent on an Accessory Device)

**Figure 3:**
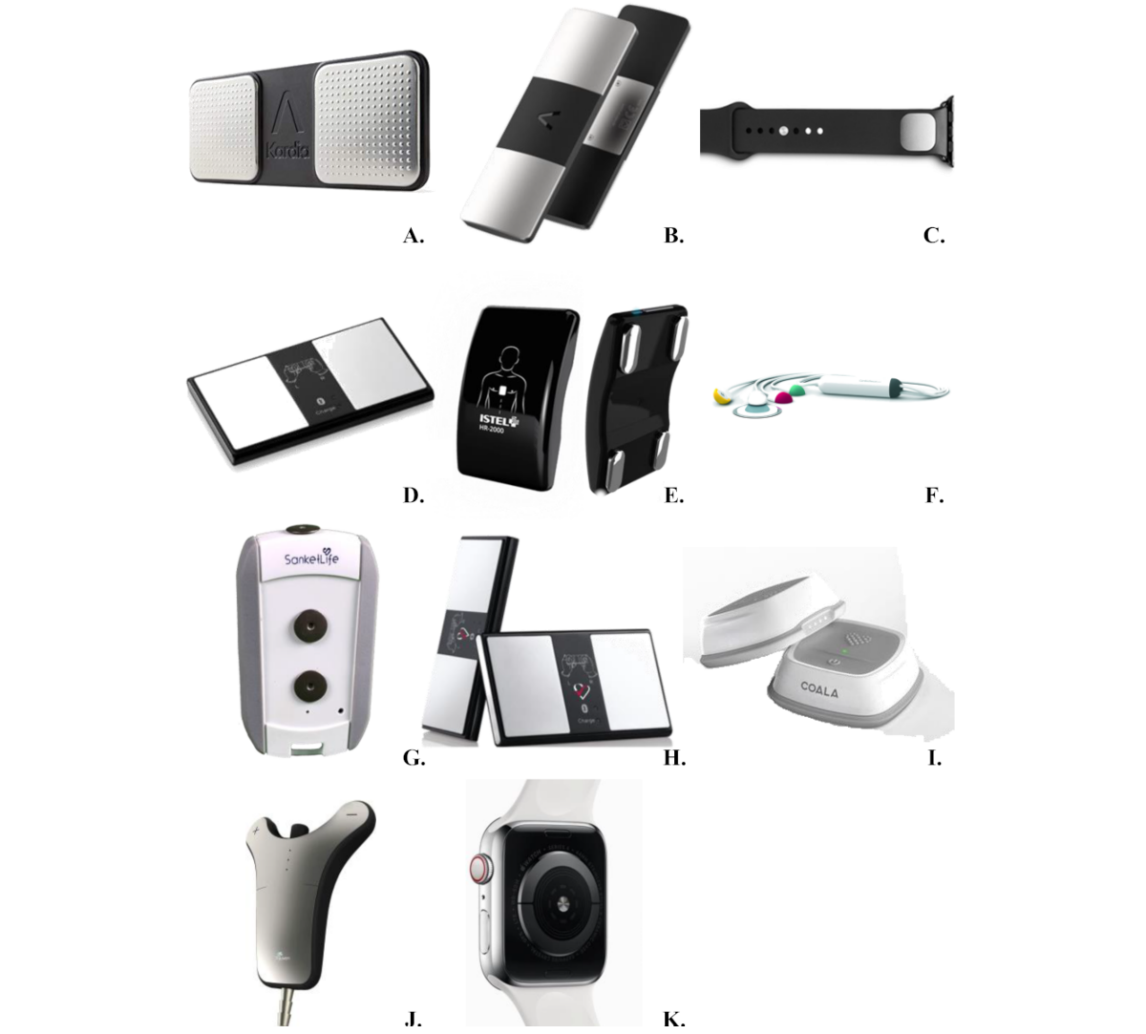
(A) Kardia Mobile (2 electrodes, 1-lead electrocardiogram). (B) Kardia Mobile 6L (3 electrodes, 6-lead electrocardiogram). (C) Kardia Band (2 electrodes, 1-lead electrocardiogram). (D) ECG Check (2 electrodes, 1-lead electrocardiogram). (E) Istel HR-2000 (4 electrodes, 6-lead electrocardiogram). (F) CardioSecur Pro (4 electrodes, 6-12-lead electrocardiogram). (G) Sanket Life-ECG, Stress, Fitness (3 electrodes, 1-12-lead electrocardiogram). (H) GEMS Mobile ECG for HeartCheck CardiBeat (2 electrodes, 1-lead electrocardiogram). (I) Coala Heart Monitor (3 electrodes, 2-lead electrocardiogram), J. i2Dtx for CardioSleeve (3 electrodes, 3-leads electrocardiogram). (K) Apple Watch Series 4 (3 electrodes, 1-lead electrocardiogram) [41-51].

Some mobile apps use ECG for recording and analyzing the signal and are dependent on accessory devices. These devices contain electrodes, whose number and location depend on whether 1-lead or 6-lead ECG is recorded. The devices examined in this paper are displayed in Figure 3, with the total number of the electrodes and recording leads shown in parentheses.

#### Kardia Mobile

Kardia Mobile (former name: AliveCor) is a clinically validated mobile device for recording 1-lead ECG and the first to be cleared by the FDA [52]. The first of 2 studies that contributed to FDA clearance of iECG was conducted by Garabelli et al [53]. The obtained ECG curve corresponds to the first (I) limb lead. The Kardia Mobile app has a built-in automatic algorithm for arrhythmia detection focused on AF.

Although some kinds of arrhythmia, like premature ventricular/supraventricular contractions or conduction abnormalities (sinus bradycardia/tachycardia, bundle branch block, or atrioventricular block) may be improperly classified as AF or even unclassified by the automatic algorithm [54,55], the app has been updated to reduce the number of unclassified traces. A study on 214 patients found the single-channel ECG to demonstrate 90.9% sensitivity (95% CI 78.3-97.5) and 93.5% specificity (95% CI 88.7-96.7) for any rhythm abnormality, and 46.4% sensitivity (95% CI 27.5-66.1) and 100% specificity (95% CI 98.0-100) for any conduction abnormality [56]. As a result, even if an automatic algorithm improperly classifies the 1-lead ECG trace as AF, it may be correctly reclassified by a clinician.

A comparison of 1-lead ECG with lead I and II of 12-lead ECG in patients taking sotalol or dofetilide found reasonable agreement between measurements of corrected QT (QTc) interval in the sinus rhythm (bias 3 ms; SD of bias 46 ms) if QTc <500 ms [57].

The efficacy of Kardia Mobile in arrhythmia detection was validated in patients with cardiovascular implantable electronic devices. A study of recordings from 251 subjects with a pacemaker (59%) or implantable cardioverter-defibrillator (41%) in paced and nonpaced states (if possible) found the readings to be adequately interpreted in 90% of paced recordings (25 of 251 recordings were “uninterpretable”) and 94.7% of nonpaced recordings (9 of 171 recordings were “uninterpretable”) [58].

Kardia Mobile is an effective tool for detecting arrhythmia or conduction abnormalities in children. It was found capable of detecting supraventricular tachycardia, AF, ectopic atrial tachycardia, atrial tachycardia, and ventricular tachycardia, and the users reported a high level of satisfaction [59]. In addition, a relationship was found between QRS dispersion and QTc intervals measured by 1-lead and 12-lead ECG in both healthy children and children with cardiac disease [60].

The QT intervals recorded by Kardia Mobile were 7 ms shorter than those from the 12-lead ECG, with only a 1.75% difference. In comparison, PQ intervals were found to be 20 ms shorter than those of conventional ECG, representing a more than 10% difference. Such a significant discrepancy between PQ intervals might lead to mimicking arrhythmias, otherwise known as pre-excitation syndrome [61].

#### Kardia Mobile 6L

The Kardia Mobile 6L is the first FDA-cleared 6-lead ECG. It has 3 built-in electrodes that record 6-lead ECG in channels I-III, aVR, aVL, and aVF [62]. The system uses the same app as the 1-lead Kardia Mobile. Thus far, it has not been included in clinical trials. It is expected that the new 6L will provide better-quality ECGs and greater information on ST-segment changes or axis determination than the standard AliveCor device.

#### Kardia Band

Kardia Band was the first FDA-cleared medical accessory for the Apple Watch Series 1 to 3 and replaced the original band. It has a specially designed band with 2 built-in side electrodes for recording 1-lead ECG [63]. The sale of Kardia Band was terminated after the Apple Watch Series 4 was released.

#### Istel HR-2000 (Istel ECG)

Istel HR-2000 (Diagnosis SA) is a CE-certified device that has 4 built-in electrodes corresponding to 5 electrodes of a conventional ECG: the left arm, right arm, left leg, and right leg. The system records a 6-lead real-time ECG and an automatic algorithm recognizes AF. High-quality reports might be analyzed by experts if the result is ambiguous. The 6-lead ECG might serve as an event recorder, thus allowing the identification of other types of arrhythmia, like supraventricular or ventricular tachycardia, premature ventricular or supraventricular contractions, and atrioventricular blocks [64]. No specificity or sensitivity values for AF detection or the correlation status between intervals measured by the device and conventional ECG has been validated in clinical trials.

#### ECG App for Apple Watch: Series 4 or Subsequent

In 2018, Apple Incorporated introduced the Apple Watch Series 4, the first smartwatch to record a 1-lead ECG, corresponding with lead I from conventional ECG. Apple Watch Series 4 included 2 black crystal electrodes on the back and another electrode that serves as a Digital Crown [65]. An Apple-sponsored multicenter study with 588 patients was performed to determine the Health app's ability to generate an ECG curve corresponding to lead I from a conventional ECG and to use an algorithm classifying heart rhythms as either a sinus rhythm or AF [66]. The results were quite promising: the sensitivity for AF detection was 98.3% and the specificity was 99.6%. Consequently, the app was awarded FDA approval for Apple Watch Series 4 and above [66].

One registered clinical trial in the Cleveland Clinic has compared to the Apple Watch Series 4 and standard telemetry monitoring [67]. Recruitment has finished, but the publication of results is still pending.

### Other Technologies

#### An App Using Phonocardiography: Heart Beat

Heart Beat is a free app that records heart rate using PCG [68]. PCG is a diagnostic technique that records cardiac acoustic phenomena [69] generated by interactions between the blood flow and heart chambers, valves, and great vessels [70]. A microphone must be placed on the chest to correctly measure the heart rate, with the surroundings remaining in absolute silence. The Heart Beat transforms the audio signal into heartbeat frequency [71]. Heart Beat has a few limitations: it has not been used in any clinical trials, absolute silence is needed when recording the signal, and its status for arrhythmia detection still remains unknown. Due to these limitations, the PCG app cannot be recommended for arrhythmia screening.

#### An App Using Seismocardiography: BeatScanner

**Figure 4:**
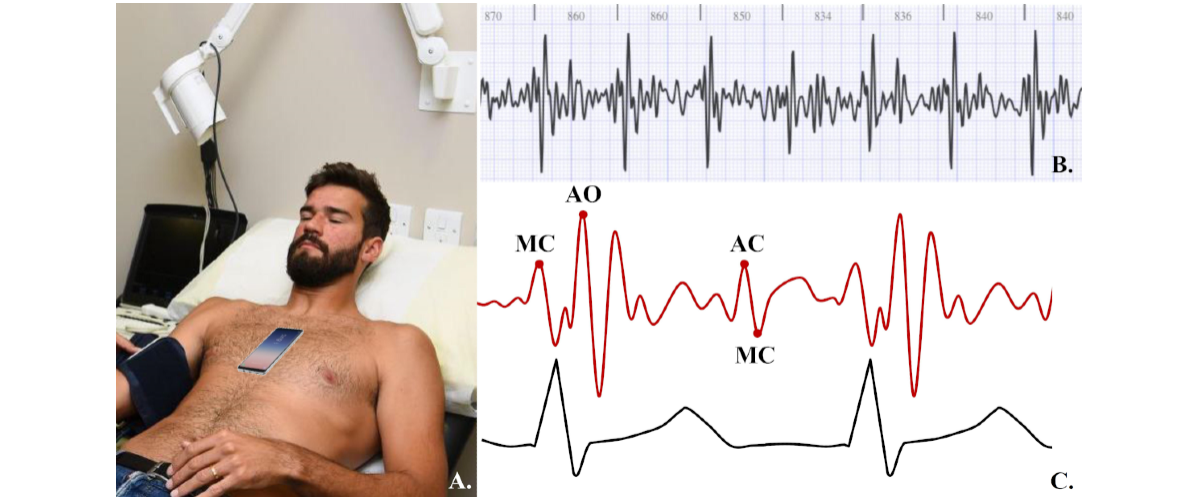
(A) The method of testing the seismocardiographic signal by smartphone (user in a reclined position or lying down). (B)_A raw seismocardiogram of a normal sinus rhythm presented in the tab "Signal representation" of the BeatScanner app [72]. (C) Correspondence between the averaged seismocardiogram (red curve) and electrocardiogram (black curve); MC describes the main peaks of seismocardiogram signal. Adapted from adapted from Shafiq et al [74]. MC: mitral valve closing; AO: aortic valve opening; AC: aortic valve closing; MO: mitral valve opening.

BeatScanner is the only app that uses SCG [72]. The app uses a very sensitive built-in accelerometer and gyroscope sensors in the smartphone to acquire microvibrations of the precordial area in reaction to heartbeats, blood flow, and respiration [73,74]. The vibrations can be studied along the superior-inferior axis (head to foot), the sinister-dexter axis (left to right), and the dorsoventral axis (back to front) [75]. The typical signal received by the gyroscope or accelerometer is called a seismocardiogram. The peaks in the seismocardiogram correspond to the opening and closing of the mitral and aortic valve [74,76]. The averaged SCG signal corresponds to ECG (Figure 4) [72,74,76]. According to Salerno and Zanetti [77], SCG might be applied to monitor the function of the left ventricle during ischemia. Paukkunen et al [78] propose that SCG may play a role in detecting atrial flutter. SCG may prove to be useful in arrhythmia detection, as the sensors are built into devices such as smartphones, and the method is noninvasive. Moreover, the sensors are cheap to develop, and the obtained signal is of high quality [75]. Unfortunately, no randomized controlled trials have compared BeatScanner with any of the methods validated for arrhythmia detection.

## Current Status and Future of Smartphone Apps in Mobile Health

A number of clinical trials have demonstrated that mobile apps both with and without accessory devices can play a valuable role in arrhythmia screening and that this role may grow in the future. The list of trials given in Table 3 includes those regarding the sensitivity and specificity of the apps and were published in PubMed before June 2020; these studies were identified by a search using the name of the app or name of technology. Some of the apps were evaluated individually (ie, FibriCheck in the DIGITAL AF II at the screening phase) so that the specificity or sensitivity is not available. Others were compared to each other or the gold standard (ie, conventional 12-lead ECG; Table 3).

**Table 3:** Mobile app in clinical research, including their sensitivity and specificity in detecting atrial fibrillation for individual applications

Study by app examined				Method with which the app was compared	Sensitivity (%)		Specificity (%)
**Smartphone apps**							
	**Photo Afib Detector**						
		Krivoshei et al, 2017 [79]	12-lead ECG^a^		87.5	95.0	
		McManus et al, 2013 [28]	12-lead ECG		96.2	97.5	
	**Cardiio Rhythm^b^**						
		Rozen et al, 2018 [32]	12-lead ECG		93.1	90.9	
		Yan et al, 2018 [33]	12-lead ECG		95.0	96.0	
		Chan et al, 2016 [31]	AliveCor		92.9	97.7	
	**Preventicus Heartbeats**						
		Brasier et al (DETECT AF PRO^c^), 2019 [34]	AliveCor		89.9/91.3/91.5^d^	99.1/98.7/99.6^d^	
	**FibriCheck**						
		Proesmans et al, 2019 [39]	12-lead ECG		96.0	91.1	
		Verbrugge et al, DIGITAL AF II^e^, 2019 [38]	N/A^f^		N/A	N/A	
	**Kardia Mobile^g^**						
		Selder et al, 2019 [54]	12-lead ECG		92.0	95.0	
		Koltowski et al, 2019 [61]	12-lead ECG		92.8	100.0	
		Himmelreich et al, 2019 [56]	12-lead ECG		87.0	97.9	
		Brasier et al (DETECT AF PRO), 2019 [34]	Preventicus Heartbeats		99.6	97.8	
		William et al. (iREAD^h^), 2018 [80]	12-lead ECG		96.6	94.1	
		Lown et al (SAFETY^i^), 2018 [81]	12-lead ECG		97.8	98.8	
		Chan et al, 2016 [31]	Cardiio Rhythm		71.4	99.4	
		Lowres et al (SEARCH-AF), 2014 [82]	12-lead ECG		98.5	91.4	
**Smartwatch apps**							
	**Preventicus Nightwatch**						
		Dörr et al, (WATCH AF^j^), 2019 [36]		AliveCor	93.7		98.2
	**Kardia Band**						
		Wasserlauf et al, 2019 [83]	Reveal LINQ		97.5	N/A	
		Bumgarner et al, 2018 [84]	12-lead ECG		93.0	84.0	
	**Health for Apple Watch Series 4**						
		Apple Incorporated, 2018 [85]		12-lead ECG	98.3		99.6

^a^ECG: electrocardiogram.

^b^Beta version not commercially available.

^c^DETECT AF PRO: Enhanced Diagnostics for Early Detection of Atrial Fibrillation–Prospective Validation

^d^Sensitivity and specificity values increased in the course of recording time from 1-3 to 5 minutes.

^e^DIGITAL AF II: Impact of Smartphone-Based Atrial Fibrillation Screening in the General Population for Primary Stroke Prevention.

^f^N/A: not available.

^g^Formerly known as AliveCor.

^h^iREAD: Assessing the Accuracy of an Automated Atrial Fibrillation Detection Algorithm Using Smartphone Technology.

^i^SAFETY: Screening for Atrial Fibrillation Using Economical and Accurate Technology.

^j^WATCH-AF: Smartwatches for Detection of Atrial Fibrillation.

Smartphone or smartwatch apps appear easy to use and are characterized by high accuracy in arrhythmia detection [86]. They may serve as noninvasive event recorders in patients with unexplained palpitations or presyncope [87]. In addition, heart rhythm monitoring based on AliveCor was well received among the pediatric population compared to conventional telemetry devices [59]. As mHealth components, mobile apps can be effectively used to detect the first episode or early recurrence of atrial arrhythmia in patients with high stroke risk and unknown AF [88] or following ablation or cardioversion [89]. Finally, screening for AF with mobile apps can lower the risk of stroke and reduce the economic burden: its use has a good cost-effectiveness ratio [82,86].

The two leading methods of arrhythmia screening are PPG and iECG, with the former being more accessible. Although PPG still needs further investigation, the results of The Huawei Heart Study [90] and The Apple Heart Study [85], conducted on 187,912 and 419,093 participants respectively, seem promising. The findings indicate that PPG may play a significant role in AF screening by detecting heart rhythm irregularity. Regarding the iECG method, European Heart Rhythm Association findings suggest that clinicians' interpretation of arrhythmia episodes detected by apps does not need to be confirmed with ECG before treatment initiation [91]. Apps based on PCG or SCG face a number of hurdles before implementation due to the substantial interference between chest sounds (in PCG) or oscillations (in SCG) with ambient sound or body tremors, the need for direct access to the chest, the need for complete contact between the phone and the chest wall, and the need for of a compulsory position to perform the measurement. These technical details make PCG or SCG less useful than iECG or PPG in everyday practice. In addition, no PCG or SCG apps have been evaluated thus far in clinical trials.

**Figure 5:**
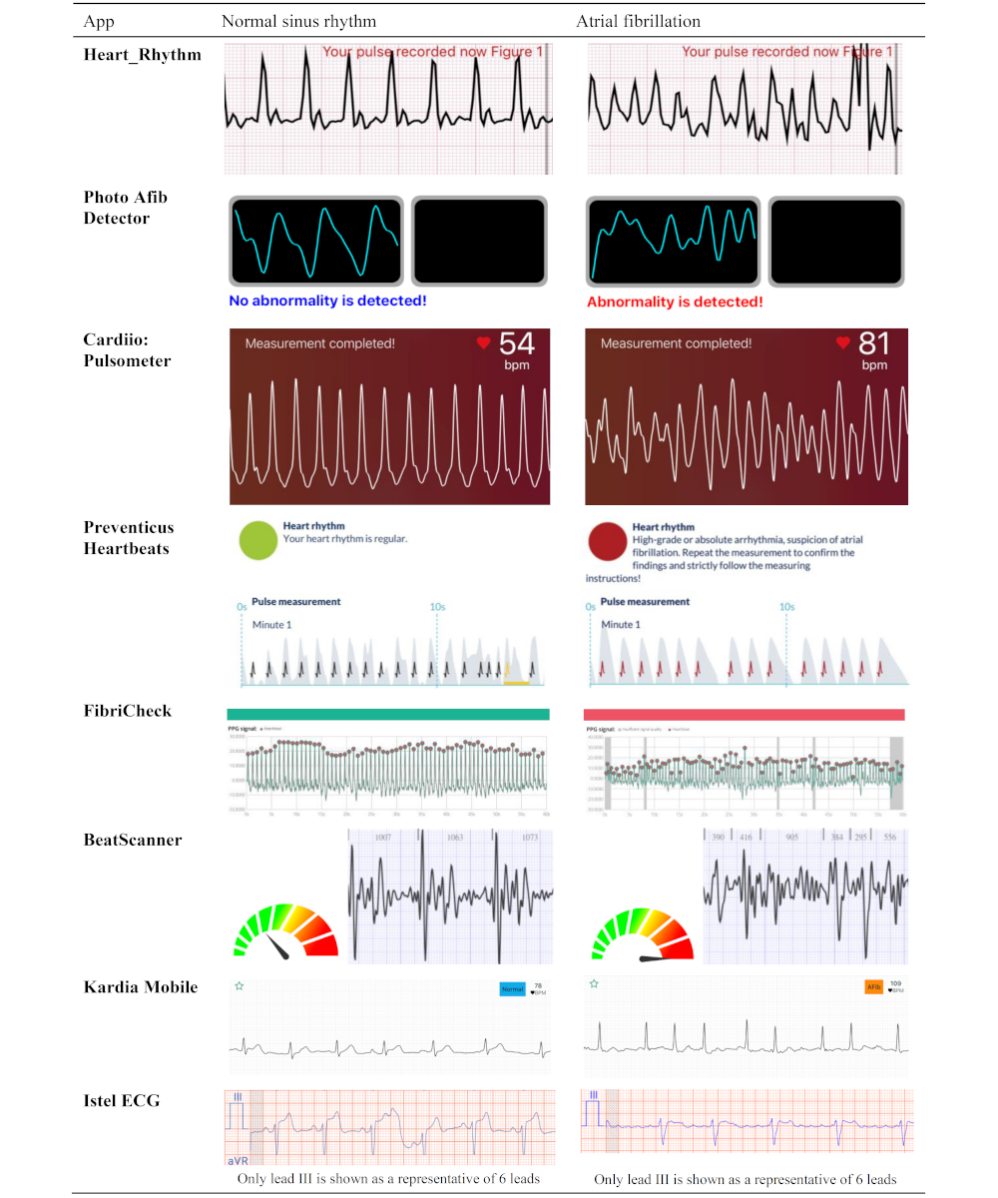
Summary of normal sinus rhythm and atrial fibrillation reports, generated by selected apps with detection of irregularity, if applicable.

A combination of technologies, such as PPG with subsets of artificial intelligence, is changing health outcomes worldwide. A summary of normal sinus rhythm and AF reports generated by selected apps is shown in Figure 5.

The role of apps supporting AF diagnosis and treatment will doubtlessly grow [90]. Since the first publication regarding the possibility of using PPG in AF detection (McManus et al [28] in 2013), its role has been developed and consolidated. Nowadays, PPG devices are not only used to confirm heart rate or check its regularity, but they can also record real-time iECG and serve as an indication for a specialist to initiate treatment [88]. The apps help detect the first episode of AF, monitor the heart rhythm in paroxysmal AF, monitor the heart rate in permanent AF, and connect the symptom with other arrhythmias or conduction abnormalities [92].

During the 2019 COVID-19 pandemic, when face-to-face consultations were transformed into teleconsultations, the value of smartphone apps and mHealth solutions in remote arrhythmia management was confirmed [93]. With the pandemic gathering pace, mobile apps will undoubtedly become a more fixed part of health infrastructure.

In addition to arrhythmia screening, some apps can be used for other applications. The literature has discussed the potential for detecting real-time myocardial ischemia using single-lead Kardia Mobile [94] or even ST-elevated myocardial infarction of the inferior wall by transforming single-lead Apple Watch Series 4 into a triple-lead smartwatch [95]. Also, the newly introduced Kardia Mobile 6L seems to be a perfect device for diagnosing myocardial ischemia and even myocardial infarction of the inferior wall, owing to its 6-lead ECG feature [62]. In addition, Yasin et al [96] found that an iECG signal could be processed to calculate the serum potassium concentration in patients undergoing hemodialysis.

## Conclusions

The rapid development of mHealth solutions may revolutionize approaches to arrhythmia screening. The ECG- and PPG-based apps demonstrate greater availability and efficacy in AF detection than those using PCG or SCG.

ECG apps can be used to detect AF; in addition, the results can also be used to precisely diagnose other types of arrhythmias (narrow or wide QRS complex tachycardia, premature supraventricular or ventricular contractions), conduction abnormalities (atrioventricular blocks, intraventricular blocks of undetermined origin), and pathological intervals (short or long QT) if the ECG trace is interpreted by a specialist. In contrast, PPG apps can be used to detect AF or to diagnose general tachycardia or bradycardia of undetermined etiology or premature contractions of undetermined origin. Therefore, it is recommended that PPG apps be used for monitoring treatment efficacy and that ECG apps be used for determining a diagnosis of AF, as robust traces are essential to starting proper treatments, such as those that included oral anticoagulants. However, due to technical details and lack of evidence, PCG or SCG apps cannot be recommended for setting a diagnosis of AF or for monitoring treatment efficacy.

As new technologies are still being developed, clinical trials of mobile apps in health care are ongoing. The findings obtained so far suggest they are on the right track to improving the efficacy of early detection of AF, thus lowering the risk of stroke and reducing the economic burden placed on public health.

## Abbreviations

AF: atrial fibrillation
CE: Conformité Européenne
DETECT AF PRO: Enhanced Diagnostics for Early Detection of Atrial Fibrillation Prospective Validation
DIGITAL AF II: Impact of Smartphone-Based Atrial Fibrillation Screening in the General Population for Primary Stroke Prevention
ECG: electrocardiogram
ESC: European Society of Cardiology
FDA: Food and Drug Administration
iECG: internet-enabled mobile electrocardiogram
mHealth: mobile health
NPV: negative predictive value
PCG: phonocardiography
PPG: photoplethysmography
PPV: positive predictive value
QTc: corrected QT
SCG: seismocardiography
WATCH AF: Smartwatches for Detection of Atrial Fibrillation
